# Application of Bamboo Plants in Nine Aspects

**DOI:** 10.1155/2020/7284203

**Published:** 2020-09-30

**Authors:** Abolghassem Emamverdian, Yulong Ding, Fatemeh Ranaei, Zishan Ahmad

**Affiliations:** ^1^Co-Innovation Center for Sustainable Forestry in Southern China, Nanjing Forestry University, Nanjing 210037, China; ^2^Bamboo Research Institute, Nanjing Forestry University, Nanjing 210037, China; ^3^College of Economy and the Management, Nanjing Forestry University, Nanjing 210037, China

## Abstract

Bamboo forests are undoubtedly one of the most abundant nontimber plants on Earth and cover a wide area of tropical and subtropical regions around the world. This amazing plant has unique rapid growth and can play an important role in protecting our planet from pollution and improving the soil. Bamboo can be used as a biofuel, food, and for architecture and construction applications and plays a large role in the local economy by creating job opportunities. The aim of this paper is to review the extraordinary tropical plant bamboo by explaining the mechanisms related to the growth and strength of bamboo and identifying ways to utilize bamboo in industry, employment, climate change mitigation, and soil erosion reduction.

## 1. Introduction

Bamboo, in the Poaceae family and the Bambusoideae subfamily [1, 2], is one of the most abundant plants in tropical and subtropical regions between 46°N and 47°S [1, 3, 4]. Bamboo can be the most important economic resource for local people of this area [1]. These woody-stemmed grass [2] species are known as some of the fastest growing plants in the world, and one native plant in Asia plays an important economic role in the livelihoods of local people living in this area [5]. Characteristics, such as fast growth, high biomass, and yield in a short time and high efficiency in few years, have allowed bamboo to be identified as a superior herb [6], which is categorized as a nontimber forest product (NTFP) plant [7]. Bamboos are used in almost 1500 commercial goods [8], which are utilized in many ways, from construction materials, food profiling, and musical instruments [5] to the production of paper pulp, fencing, basketry [9], water pipes, utensils [10], bicycles [11], bridges [12], and low-rise housing [13]. According to the FAO in 2010, bamboo covers more than 31 million hectares of forestland around the world, and more than 60% of it is located in China, Brazil, and India [14], while it is abundant in other countries on three continents, namely, Asia, Latin America, and Africa; moreover, bamboo covers more than 0.8% of the forest area in the world [15]. Generally, 80% of bamboo forests are in Asia, 10% in Africa, and 10% in Latin America [16]. In the world, bamboo contains 1225–1500 species in approximately 75–105 genera [17]. Among these countries, China, with more than 500 species in 39 genera, is one of the countries with native bamboo, which is called “The Kingdom of Bamboo” [18], where bamboo covers more than 6.01 million hectares of China's forests [19]. This amazing herb famously has different local names in Asia and is called “friend of people,” “wood of the poor,” and “the brother” in China, India, and Vietnam, respectively [20, 21]. One of the most important features of bamboo is the rapid rate it reaches maturity, which can be three years, while other woods need approximately 20 years to reach maturity. The bamboo growth rate is also stunning; in some reported cases, it is approximately two inches per hour, and the height can reach 60 feet in only 3 months [22]. All these reasons have led to an increase in Chinese bamboo forests from 4.21 to 6.01 mil·ha (43%) from 1998 to 2013 [23]. Bamboo has great potential for use in construction because it has nodes, which improve bending and tensile strengths and can be compared with steel and cement [24]. Bamboo is a renewable bioresource that can have a short period of growth with a high CO_2_ fixation rate [25]. Bamboo can absorb approximately 3.73 cubic meter of CO_2_, which means it can absorb the equivalent of carbon dioxide emissions from approximately 2 cars in one day and 1.83 kg carbon in less than one month, so it can be a good option for reducing global warming and climate change [26]. Bamboo is one of the most economical forest plants, and new applications of bamboo are found every few years. In recent years, the entry of bamboo into the textile industry has created antibacterial and UV absorption bamboo clothing, which is caused by a characteristic of lignin in the bamboo fiber [27, 28]. One experiment on the removal of two bacteria, *S*. *aureus* and *E*. *coli*, showed that the use of the bamboo fiber led to the maintenance of 88% of the antibacterial properties after 20 washes, as well as anti-UV properties, which increased from 8.16 to 18.18 when using bamboo pulp fibers [29]. In general, today's bamboos play a considerable role in human life, and they cover a wide range of human needs from environmental protection to use as home appliances. The aim of writing this paper is to identify the most commonly utilized bamboo for researchers by describing the mechanisms available in this unique plant. Below are the most important ones.

## 2. Bamboo Is Uniquely Tall and Fast Growing

Bamboos belong to the Poaceae (Gramineae) family, and they are known to be a fast-growing and the tallest species in this family [30, 31]. The bamboo rhizomes in bud sites lead to the emergence of new bamboo shoots, which expand into a new culm [32]. Bamboo culms emerge in spring, while bamboo root systems and rhizomes expand throughout the year, but growth will increase during the summer and autumn [33]. Culms are divided into nodes, and nodes are separated from each other by internodes [34]. In bamboo, growth stages have three steps, and they are made with changes in the cell's structure, which include division, expansion, and hardening of cell walls [35]. According to the definition of these 3 steps in the bamboo life cycle, cell division is related to regulation of hormone interaction between plants, while in the cell expansion cycle, cells can be expanded with the process of cellulose synthesis by turgor pressure. In addition, secondary cell wall deposition leads to hardening in cell walls [36]. On the other hand, in addition to cellular processes, bamboo elongation is dependent on physiological structure, such as lignification. The lignification process is different for various plants, but it generally has 3 mechanisms in the stem, which include the polymerization of lignin precursors, transport, and biosynthesis. The identification of distribution and content of lignin is important to determine the critical period of bamboo elongation and biomass. The results of one study indicated that when the content of lignin in the culm reaches half of the mature culms at the end of June, growth elongation became complete [37]. However, the most important reason involved in the explosive growth of bamboo is related to nonstructural carbohydrates (NSCs). Generally, the main products obtained by photosynthesis are soft carbohydrates (SCs) and nonstructural carbohydrates (NSCs), of which SCs are composed of pectin hemicelluloses, lignin, and cellulose but NSCs include starch and soluble sugars. NSCs are large and as a source of carbon play a vital role in exploring the period of time of bamboo shoot growth when it cannot provide carbon independently. In one study, it was shown that when shoots are growing, NSCs are simultaneously being transferred from their branches, leaves, rhizomes, and trunks to shoots, and this transfer stops when young shoots obtain enough photoassimilates and enough carbon [38]. The rate of bamboo growth in the culm is different and dependent on species, but it can be from 9.7 to 24.5 cm·d^−1^ for *Bambusa oldhamii* (synonyms *Leleba oldhami*) and *Phyllostachys makinoi* [39], respectively, to more than 100 cm·d^−1^ for *Phyllostachys edulis* [40]. This range of culm growth in different bamboo species can be between 7.5 and 100 cm^−1^ [41].

## 3. Bamboo Protects O_2_ and CO_2_ on Earth

Bamboo plants, with more than 40 million hectares around the world, are one of the most important plants in improving climate change due to the high bamboo biomass stocks and carbon storage [42]. Bamboo can sequester and capture atmospheric carbon within its lifespan, which can offset CO_2_ emissions by storing high concentrations of CO_2_ in the hollow parts of bamboos. CO_2_ effluxes have been reported from culm, buds, and nodes [43]. Many studies have reported the role of bamboo forests in global carbon cycling [44–49]. Among bamboo species, moso bamboo, which represents 75% of all bamboo forest area in China [19], has been known as a carbon sink and has a high ability for carbon sequestration [50–52]. Carbon in the bamboo rhizome system can be transferred to new culms and aerial organs [53,54]. The average amounts of carbon stored by forests in China and the world are 39 mg·C·ha^−1^ and 86 mg·C·ha^−1^, respectively, while this average in bamboo forests in China is 169–259 mg·C·ha^−1^, which revealed the bold role of carbon stocks of bamboo species in China [55]. Bamboos are known to be successful plants at absorbing wastewater from agriculture, industry, animal breeding, and pollution, which can be related to the neutral characteristic in resistance to stresses. Bamboos, through their phytoremediation potential, can clean up polluted soils and can also accumulate silicon in their bodies to alleviate metal toxicity, and this accumulation in nature is up to 183 mg·g^−1^ of SiO_2_ [56]. In one experiment on the efficiency of three bamboo species on wastewater removal over 2 years, the results showed that the soil-bamboo system could remove 98% and 99% of organic matter and nutrients, respectively [57]. Therefore, bamboo is a great recommendation for decreasing the negative effects of climate change and a big sink of carbon in nature, which plays an important role in adjusting and improving human ecosystems [58].

## 4. Bamboo Binds the Soil

Bamboo plays a protective role in decreasing soil degradation, including the reduction of biodiversity, soil nutrient depletion, and soil erosion [59–61]. In one study in a long-term monitoring experiment of bamboo, planting revealed that bamboo can decrease topsoil erosion in sloping croplands [62]. However, the intense management of bamboo has a negative effect on the soil microbial functional diversity and soil microbial activity, which are indicators of soil quality [63]. However, the results of other studies reported that bamboo as a fine biochar had a positive impact on increasing the microbial community related to size, impacting C cycling by decreasing their soil enzyme activity and led to increasing (higher) CO_2_ emissions [64]. On the other hand, the lack of right management in the annual harvest of shoots and timber for economic purposes led to a decreasing rate of output nutrients to input nutrients in the soil, which, according to the different structures of bamboo compared to other forest plants with high nutrient absorption, can convert forest soil to poor soil [58]. Many studies have reported that the biochar is a good application for emendation and decontamination in soil [65–69]. Additionally, with some mechanisms, such as increasing pH in soil, the biochar can lead to the immobilization of heavy metals such as Cu, Cd, Pb, and Zn in the soil [70, 71]. In one study, Wang et al. indicated that bamboo as a biochar can reduce mobile fractions of some heavy metals, such as Cd, Cu, Mn, Ni, and Zn, in soil and enhance the physiological efficiency in soybean exposed to soil contamination by increasing the number and weight of nodules of soybeans in contaminated soil [72], which has shown that bamboo species have phytoremediation potential to detoxify soils contaminated with heavy metals with the characteristics of high metal tolerance and extreme biomass production [73]. Bamboo charcoal has an important role in adjusting soil pH, enhancing nutrient absorption, and improving soil structure [74]. Additionally, bamboo, as a natural material, can improve the ductility and strength of the soil structure. In one study with a combination of bamboo chips with cement, the results showed that bamboo could increase erosion resistance and improve soft ground [75]. In general, studies have shown that bamboo can play an important role in improving the soil structure or can bind to the soil.

## 5. Bamboo Is Strong

As a nontimber plant, bamboo is popular worldwide and rapidly produced [76]. Bamboo, because of microfiber structures with lignin and hemicellulose (lignin-carbohydrate complex (LCC)), has a greater strength than concrete and steel by weight [77], and this strength is due to the thickness of the fiber in the sclerenchyma tissue [78]. The diameter of the fibers at the site of the nodes is another factor in the stiffness and bending of the bamboo, so that the fibers wrapped in it hold [79]. It prevents the generation of endless bamboo yarns [80]. At present, the diameter of these fibers in this region is between approximately 90 and 250 *μ*m, which itself is a resistance factor against bending in bamboo [79]. Additionally, bamboo, because of low density (1.4 g/cm³) and high mechanical characteristics, can show high tolerance against pressure and bending [79]. The results of some studies have reported that the strength of bamboo is related to thickness, diameter, moisture content, and density, which increase with age, so that the age between 2.5 and 4 years has optimal strength, and then it will decrease after this age [81, 82]. One of the most important cases that indicates the strength of bamboo is the use of bamboo in scaffolding. For many years, bamboo has been used as scaffolding in the construction industry in Hong Kong and Southeast Asia. Starting 2000 years ago, bamboo scaffolding was considered to have characteristics such as an increase in safety from the practical experience of workers, resistance to moisture, low cost, high adaptability, and for a short period of time, it has been used in the south of China, Hong Kong, and other countries in this area [13]. According to the above results, it is concluded that bamboo is one of the strongest tropical plants, with comparable strength to cement and steel.

## 6. Bamboo Is Flexible

Flexibility and fracture toughness of bamboos come from the special cellular material in these plants [83]. Bamboo structure consists of fiber, which covers internal structures such as vascular bundles of parenchyma cells and the epidermis [83, 84]. They are also pathways for the growth of the cracks in longitudinal and radial directions [83]. Epidermis, as thick sheaths, surround bamboo, while vascular bundles with longitudinal tissues play an important role in the transport of water and nutrients in the bamboo body by organs such as vessels and phloem. On the other hand, other parts are occupied by aerenchyma. However, all of these structures are covered by unidirectionally oriented fibers [85], which include 40% of a bamboo culm [86]. Bamboo fiber is mainly (90%) three parts, including lignin, cellulose, and hemicelluloses, which have an important role in mechanophysical characteristics of bamboo in flexural strength [22] and are related by chemical linkage and physical binding [87, 88]. Therefore, lignin, hemicelluloses, and phenolic acids are involved in the strength of concentrations and covalent bonding in layers of the cell wall [22], and this bonding, in addition to increasing the mechanical strength, can lead to the resistance of the cell wall to biological degradation and can be vital for the rigidity of lignin in the cell wall [89], leading to the flexible character in bamboos.

## 7. Bamboo Biofuel

Many studies have reported that bamboo, as a forest product, has potential for use as a biofuel, along with other woody plants [74, 90–92]. Bamboo, because of the high amount of sugar, is known to be a suitable plant for a feedstock of chemical products, such as lactic acid and fuel ethanol [93]. It can also be used as biogas [94]. Bamboo, as a fast-growing plant with a high yield of lignocellulosic biomass in short time, is considered a good option for use as a biofuel [95], such as bioethanol, by the top holocellulose content (high dry weight of more than 70%) [74, 92, 93, 96]. Lignocelluloses have abundant sugar resources such as pentose and hexose and can be converted to fuel alcohol [97, 98]. Moreover, bamboo biomass has key characteristics such as low lignin and high cellulose contents and is known to be a suitable material for the production of bioethanol [99]; moreover, the possibility of obtaining bioethanol from the SPS hydrolysate of bamboo has been shown [100]. It has reported that there is a possibility of extracting 143 L of ethanol in each dry ton of bamboo [99]. On the other hand, the process of producing 1 kg ethanol requires 8.5 kg of sulfuric acid, 65.8 L of process water, and 6.2 kg of bamboo [100]. Additionally, because of important characteristics such as the alkali index and low ash content [101], bamboo can be a good alternative for other woody plants for biofuel purposes [102]. However, bamboos need pretreatments, such as an alkaline peroxide treatment to remove rigid lignin, which covers holocellulose components, and these pretreatments can also optimize enzymatic saccharification for the production of sugars [93]. On the other hand, bamboo culms are known as resources of bioenergy, which in one experiment, showed that young culms are suitable for bioconversion process [103]. Among biofuels, butanol is important because of the ability to produce higher energy without blending with gasoline and the ability to transport it in existing gasoline pipelines [104]. In addition, the energy content is higher than that of ethanol [105]. The results have shown the high temperature, acid concentration, and time can increase the sugar yield of bamboo, which is obtained by conversion of lignocellulosic biomass in bamboo species to butanol [106]. Generally, bamboo can be used as a biofuel and for bioenergy.

## 8. Bamboo Is Beautiful (Used in Architecture)

Bamboo, as a green and sustainable material, has an important role in new architecture, so that in the future, architecture based on green building will be built with bamboo as one of its most important materials. In this case, bamboo is very familiar among scientists because of its energy savings, zero fossil emissions, and environmentally friendly nature [107]. A simple comparison of the strength of joints in grains with bamboo joints shows that strength perpendicular to joints and strength parallel in joints in bamboo is 45% and 8% higher than the grains in internode parts [108]. Based on the growth factor, bamboo is one of the best options for wood products [16]. Bamboo, despite having some disadvantages, including the difficulty of modeling due to hard tissue, a rough texture, and rugged material properties, it still is important for design purposes because of some characteristics such as water resistance, bending resistance, hardness, and environmentally friendly nature [109]. Bamboo timbers are luxury woody material used in furniture, flooring, and architecture [110]. Among fibers, bamboo is useful because it is an abundant tropical plant, and its material distribution, microstructural shapes, low cost, and easy accessibility make it an excellent material to build woody houses throughout the world [111]. Bamboo scrimber, which is produced during processes such as exposing bamboo to hot dry air, has been reported as a good option for use in outdoor landscaping, garden furniture, decoration, and civil engineering. There are several reasons for this: the enhancement of water absorption, width swelling, and thickness in bamboo scrimber [112]. Recent studies have shown that bamboo combined with reinforced concrete can increase building (construction) resistance to earthquakes, which can be an important benchmark for the use of these forest resources in earthquake-prone areas [113]. The external resistance of bamboos, such as compressive, tensile, and static bending strength, shock and shear resistance, and elastic properties, is related to elements in bamboo including bamboo stalk parts, moisture content, and type of bamboo [108]. These properties in bamboo are much greater than those in woods, so that the compressive and tensile strength of bamboo are 20% and 2 times more than those of woods [107]. Bamboo, as an agricultural crop, has great potential for use in the design industry and polymer composites [114], which are identified as a natural engineering material [22].

## 9. Bamboo Is Edible (Using Bamboo Shoots as Food)

From a long time ago, bamboo shoots have been a tasty food with a high fiber content and have been eaten by the local people in southern Asia, especially in China [63,110]. Bamboo shoots are powerful sources of fiber, known as dietary fiber, with low fat and calorie contents [115]. Bamboo also has necessary amino acids, potassium, antioxidants, selenium [116], vitamins, carbohydrates, and protein. However, the Bamboo Age Index is important; in one experiment, Nirmala et al. reported that the amount of vitamins and the mineral content decrease with increasing age of bamboo [117]. Thus, young bamboo culm can be a resource for fiber and starch, which can be used for food applications, such as bamboo flour, pasta, meat products, cheese, yogurt, and bread. Additionally, it contains abundant phytosterols and dietary fiber, and it can be found in the commercial market as canned food [118]. The bread produced by yeast from bamboo shoot is of high quality. In one experiment, yeast from bamboo shoots was shown to have the highest specific volume with a high moisture content in the content of crust and crumb compared to other commercial yeasts, which makes the bread much softer and brighter and increases the quality of the bread [119]. Bamboo shoots are also considered for medical purposes for the treatment and control of cholesterol and diabetes from different products obtained from bamboo shoots, such as bamboo salt and bamboo vinegar [116]. On the other hand, in addition to humans, bamboo shoots are beneficial and tasty food for animals. Bamboo shoots are a source food for some rare animals such as African golden monkeys (*Cercopithecus mitis kandti*), mountain gorillas (*Gorilla beringei beringei*) [120], and especially panda, which guarantees the survival of the panda generation [121]. In general, bamboo, as a beneficial plant with plenty of fiber, plays a considerable role in the food chain of humans and especially in animals.

## 10. Bamboo Presents Opportunities

Bamboo has been known as “poor man's timber” because more than 20 million tons of bamboo is often collected in rural areas by local people, which plays an important role in the local economy [122]. In China, there are approximately 200 species and 16 categories of bamboo cultivated for economic and ecological purposes [107]. Bamboo planting worldwide is approaching 220,000 km^2^, which produces 15–20 million tons of products annually [123]. It has been estimated that approximately US$2.5 billion of international trade is related to the bamboo industry every year, which directly or indirectly has provided 2.5 million jobs around the world [16]. Moso bamboo, as one of the largest species in Asia, has a share of US$5 billion in China's forest product industry each year [124, 125]. Based on the reports of the State Forestry Administration of China in 2012, bamboo products have shown a significant increase of approximately US$19.7 billion [126]. There is one small market of bamboo, which is called the traditional market of bamboo, that directly provides income to local people, and these market products include chopsticks, handicrafts, bamboo shoots (food), and medicine. However, often bamboo businesses have been obtained by emerging markets, which use the woody timber of bamboo for flooring, roofing, construction, architecture, and furniture, which makes it responsible for almost 3–7% of the timber trade in the tropical and subtropical areas [122]. In general, all these statistical reports represent the important role of bamboo in local economies, as well as providing job opportunities.

## 11. Conclusion

Bamboo is known as an ancient grass with woody timber that covers 1–3% of all tropical and subtropical areas. Bamboo has many uses, mainly in construction (flooring, roofing designing, and scaffolding), furniture, food, biofuel, fabrics, cloth, paper, pulp, charcoal, ornamental garden planting, and environmental characteristics, such as a large carbon sink and good phytoremediation option, improving soil structure and soil erosion. Bamboo has the highest growth rate of all tropical plants. After emerging as a shoot, bamboo can complete the growing process in both diameter and height in 35–40 days. The growth rate has been observed at up to one meter per day, that is, approximately 2.5 cm per hour. This extraordinary power of growth is due to the bouncy properties of the nodes and the intracellular structures of internodes. For thousands of years, bamboo has been an economic source of livelihood and a natural workshop for the employment of local people. However, in recent decades, this economic resource has expanded out of its border and has led to the creation of jobs for many people around the world, such that it has provided 2.5 million jobs around the world. Bamboo as a green and sustainable material plays an important role in new architecture, so that in the future, architecture based on green building will be built with bamboo as one of its most important materials. In this case, bamboo is very familiar among scientists because of its energy savings, zero fossil emissions, and environmentally friendly characteristics. The ability to use bamboo as a timber wood, with special characteristics such as being lightweight, low in cost, and having high performance, makes it a green material in construction and architecture. Flexibility and fracture toughness of bamboos come from the special cellular material in these plants. Bamboo protects the planet. Bamboo forests can reduce the negative effects of global warming so that bamboo can store and absorb carbon and CO_2_ in its organs, and as one phytoremediation option, it can also detoxify environmental contaminations. Bamboo binds the earth so that bamboo as a biochar can improve soil structures; thus, bamboo has a protective role in decreasing soil degradation, including the reduction of biodiversity in soil nutrient depletion and soil erosion. Because bamboo has a high yield of lignocellulosic biomass in a short time, it is considered a good option for use as a biofuel. Bamboo shoots, as a tasty food with high fiber content, have been eaten by local people in southern Asia, especially in China. Additionally, bamboo products obtained from the bamboo shoots are used in traditional medicine to control many diseases, including diabetes and cholesterol. So, all of these factors indicate the importance of recognizing this tropical plant. It seems that the most bottleneck problems existing in bamboo are related to lack of awareness of bamboo potentials and as well as a lack of enough attention to the development of marketing in this sector. So, the governmental organizations and national campaigns can help to raise awareness about bamboo. In this regard, it can be used from the experiences of leading countries in this field such as China. Due to the high demand for the use of environmentally friendly green products, the global bamboo market is expected to grow substantially in the near future. On the other hand, use of bamboo woods as one low-cost construction material encourages countries to use bamboo in the development of cities and villages, which can greatly contribute to the development of the bamboo trade in the world. The authors' goals for authoring this review article are to describe the uses of bamboo plants in today's life, clarify some of the mechanisms involved in bamboo growth and strength, and recall the key role of bamboo plants in improving climate change and global warming that have not been commonly mentioned.
